# Melamine-Based Molecularly Imprinted Monoliths Targeting Glyphosate in Aqueous Media: Synthesis and Binding Mechanism Elucidation

**DOI:** 10.1021/acsomega.4c06690

**Published:** 2025-05-23

**Authors:** Chau Minh Huynh, N. Tan Luong, Trung Nguyen, Ngoc Phuoc Dinh, Jean-François Boily, Knut Irgum

**Affiliations:** † Department of Chemistry, 8075 Umeå University, S-90187 Umeå, Sweden; ‡ Diduco AB, Tvistevägen 48C, S-90736 Umeå, Sweden

## Abstract

Cross-linked melamine imprinted monoliths targeting glyphosate were synthesized using 4-phosphonobutanoic acid (PBA) and *N*-(phosphonomethyl)­iminodiacetic acid (PMIDA) as templates. The binding capacities, evaluated in an aqueous medium, showed that both PMIDA and PBA promoted selective binding sites with imprinting factors of 2.5 and 1.7, respectively. Despite a relatively low imprinting factor, the polymer imprinted with PMIDA showed a noticeably higher binding efficiency in the presence of sodium chloride compared to the nonimprinted reference, demonstrating an ability to selectively target the desired analytes in real sample matrices. Spectroscopic investigations using Fourier transform infrared and ^1^H nuclear magnetic resonance spectroscopy revealed the formation of “memory pockets” for glyphosate molecules in the imprinted melamine–formaldehyde scaffold promoted by simultaneous contributions from (i) hydrogen bonding with N–H/O–H moieties and (ii) electrostatic interaction toward the triazine ring.

## Graphical abstract

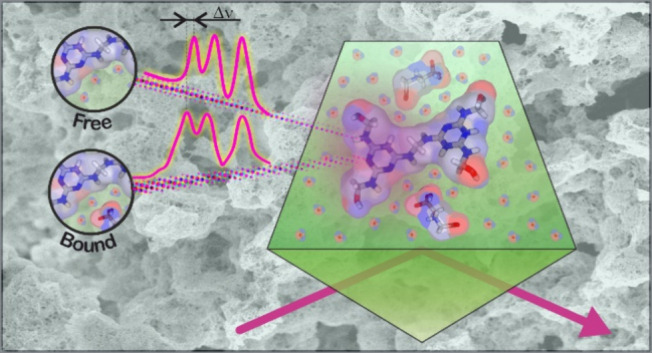

## Introduction

Glyphosate, an organophosphorus-based herbicide, has been widely used in agriculture for several decades. Its efficiency in controlling weeds and enhancing crop yields has contributed significantly to food production and security. However, the accumulation of glyphosate and its degradation products in water bodies and soil has led to substantial concerns about environmental and human health risks.^1–3^ The monitoring and control of glyphosate in agriculture are, therefore, a crucial task. However, glyphosate is a difficult analyte due to its extreme hydrophilicity and ill-suited properties for chromatographic separations.^4^ One of the most common approaches for determination of glyphosate therefore involves a sample derivatization step, which is time-consuming and requires extra instrumentation.^5–9^ Since glyphosate has both carboxylate and phosphonate groups, partitioning onto solid matrices has been used to retain it from aqueous matrices, notably by anion exchange^6,10,11^ or by Lewis acid–base interactions on inorganic oxide materials, such as alumina-coated iron oxide^12^ or titanium dioxide,^13^ or on Pd­(II)-loaded ion-exchange resins.^14^

One of the recent efficient approaches is the use of molecularly imprinted polymers (MIPs), in which the carboxylate and phosphonate moieties are the key binding epitopes. MIPs based on aminoethyl methacrylate and acrylamide have hence been used to enrich glyphosate and its derivatives from aqueous solution.^15–19^ The binding between glyphosate and the polymer backbones results from a combination of hydrogen bonding and electrostatic interactions. Moreover, high-nitrogen-content polymer backbones and receptors using pyrrole^20,21^ or urea-based monomers^22–27^ have been investigated for glyphosate binding. Results of Kadam et al.^24^ and Rouhi et al.^25^ on multidentate bis-urea-based synthetic materials suggest that both phosphonate and carboxylate moieties are involved in the interaction through hydrogen bonding. If hydrogen bonding is the primary contributor to interactions, an increased water content in the adsorption medium will not only cause a strong decrease in binding affinity but also exert a positive effect on glyphosate binding by hydrophobic interactions involving the methylene bridges. Moreover, the affinity of glyphosate toward bis-urea-based MIPs has been enhanced by the introduction of positively charged moieties on the polymer backbone, e.g., quaternary ammonium ions, protonated amines, or Na^+^ complexed by crown ethers.^23^

The basic concept of molecular imprinting is based on the formation of a three-dimensional cross-linked polymer network in the presence of a template molecule, the purpose of which is to establish energetically favorable interactions between functional groups of the targeted compound and functional monomers of the growing polymer system.^28,29^ As long as the polymer network is flexible, the functional groups will tend to rearrange in the reaction cocktail to form a polymer backbone minimizing the internal energy.^30^ Among the most important factors to establish MIPs with high and specific affinity is therefore the interaction between the template, which should have an epitope also found in the target molecule, and the generally polar functional monomers of the polymerization mixture, promoting electrostatic interaction, hydrogen bonding, or other polar interactions. The strengths of these interactions upon binding the targeted molecule could be monitored by spectroscopic methods, such as UV,^31^ FTIR,^32^ and NMR.^23,33–35^ By optimizing the ratios of functional monomer(s) to template, high-affinity MIPs can be produced in a time-efficient way.

This study focuses on (i) the preparation of porous monolithic MIPs targeting glyphosate in a water medium and (ii) evaluation of the binding mechanisms of the resulting MIPs using a combination of batch rebinding and spectroscopic approaches. Imprinted monoliths based on cross-linked melamine–formaldehyde scaffolds can be synthesized in water and show molecular recognition in aqueous matrices.^36–41^ Both the functional monomer melamine and the cross-linker formaldehyde have hydrophilic groups, notably amino and hydroxyl functionalities, with more of the latter being converted to ether linkages during the polymerization. These functionalities render the polymeric material hydrophilic and compatible with aqueous samples. Our previous works^36,37^ have confirmed that molecularly imprinted cross-linked melamine–formaldehyde monoliths can be prepared that have selectivity toward phosphonate and carboxylate groups in aqueous media. Cross-linked melamine–formaldehyde should therefore be a good candidate as a polymer scaffold for molecular imprinting targeting glyphosate. This work hence focuses on the preparation of MIPs with selectivity toward glyphosate, coupled with the use of proton nuclear magnetic resonance (NMR) titration to evaluate the formation of the monomer–glyphosate complex and Fourier transform infrared (FTIR) spectroscopy to elucidate the binding mode of MIPs in aqueous medium at varying electrolyte concentrations.

## Materials and Methods

### Reagents and Materials

*N*-(Phosphonomethyl)­glycine (glyphosate, >95%) was purchased from Biosynth AG (Staad, Switzerland). *N*-(Phosphonomethyl)­iminodiacetic acid hydrate (PMIDA, 95%), poly­(propylene glycol) with an average molecular weight of 4000 Da (PPG4000), 1,3,5-triazine-2,4,6-triamine (melamine, 99%), poly­(melamine-*co*-formaldehyde) methylated (PMFM, average *M*_n_ ≈ 432; 84 wt % in 1-butanol), heavy water (D_2_O, 99.9%), and deuterated acetonitrile (ACN-*d,* ^3^ > 99.8 atom-% D) were from Sigma-Aldrich (Steinheim, Germany). Polyoxymethylene (paraformaldehyde; “extra pure”) was purchased from BDH Chemicals (Poole, UK), and 4-phosphonobutanoic acid (PBA; 98.0%) was from Tokyo Chemical Industry (Tokyo, Japan). Sodium carbonate (99.5%), sodium hydrogen carbonate (99.7%), acetonitrile (ACN, “analytical grade”), and formic acid (FA, 98–100%) were from Merck (Darmstadt, Germany). The poloxamer Pluronic F127, an α,ω-hydroxy-poly­(oxyethylene)-*block*-poly­[oxy­(1-methylethylene)]-*block*-poly­(oxyethylene) triblock copolymer [EO_99_PO_69_EO_99_; MW ≈ 12,700] was from BASF (Ludwigshafen, Germany). Sodium chloride (99.0%) was purchased from J.T. Baker (Radnor, PA, USA), and sodium hydroxide (98.8%) was from Akzo Nobel (Bohus, Sweden). Water was prepared by a Milli-Q system from Merck Millipore (Burlington, MA, USA) and checked for conductivity close to 55 nS/cm at 25 °C.

### Preparation of Molecularly Imprinted Monoliths

A precondensate was prepared according to our previous description^36,37^ by adding 8.580 g of melamine and 6.000 g of paraformaldehyde to a 100 mL round-bottom flask, followed by 48.000 g of water to create a suspension. The flask was then placed in an oil bath preheated to 80 °C. After approximately 25 min, the suspension turned into a transparent solution, which was rapidly cooled to room temperature and used within 4 h. A porogen solution was separately prepared by dissolving 1.800 g of PPG4000 and 16.800 g of Pluronic F127 in 180 mL of acetonitrile. This porogen solution was stored at ambient conditions until fully utilized and was given a 30 s treatment in a sonication bath before each use.

The final step consisted of weighing the template (PBA or PMIDA) into a glass vial containing 6.000 mL of porogen solution, followed by adding 7.800 mL of precondensate. If needed, 350 μL of formic acid was thereafter added. The vial was then stirred to prepare a well-mixed homogeneous solution. The vials were thereafter cooled to −20 °C, at which temperature the polymerization was allowed to proceed for 4 days. The NIP monolith was prepared in the same way but without a template added (Table 1).

**Table 1:** Chemical Compositions Used in the Syntheses of Imprinted Monoliths

	monolith designation				
chemical/mixture	NIP	M-PBA0	M-PBA1	M-PMIDA0	M-PMIDA1
precondensate	7.800 mL	7.800 mL	7.800 mL	7.800 mL	7.800 mL
porogens	6.000 mL	6.000 mL	6.000 mL	6.000 mL	6.000 mL
PBA		309.8 mg	309.8 mg		
PMIDA				418.6 mg	418.6 mg
formic acid	350 μL		350 μL		350 μL

After the reaction, the materials were recovered by cracking the vials open, and the monolithic materials were broken into small pieces (2–3 mm) and packed into an RBR S2 rotating bed reactor (SpinChem AB, Umeå, Sweden) for cleaning. The packed RBR was then rotated at 500 rpm in a baffled vessel containing 250 mL of 500 mM aqueous NaCl for 2 h. In this step, chloride ions (Cl^–^) at high concentrations would compete with the electrostatic interaction between the template molecules (PBA or PMIDA) and the polymeric backbone. Water molecules would also weaken hydrogen bonding. This was repeated three times before repeated washings were performed with fresh water in the same manner until the washing solution conductivity was <5 μS/cm. Finally, the materials were washed three times with methanol before drying in a vacuum oven at 40 °C overnight, followed by crushing and dry sieving using 400 and 200 mesh sieves with openings of 37 and 74 μm, respectively, prior to further characterization.

### Affinity Test for Glyphosate on Imprinted Monoliths

Five milligrams of materials was suspended and shaken in 1.000 mL aliquots of glyphosate in water at varying concentrations (0, 50, 100, 300, 500, 1000, 1500, 2000, 3500, 5000, 7000, and 10,000 μM) for 20 h on an IKA Orbital Shaker (IKA-Werke, Staufen, Germany) at room temperature. The concentrations of unbound glyphosate in the supernatants were then analyzed by an ion chromatography method. The amount of bound glyphosate per unit surface area of polymer (*B*) was calculated according to

$$B=\frac{(C_{0}-C)\times V}{m\times S}$$ 1

with *C*_0_ and *C* being the glyphosate concentrations of the initial solution and the supernatant, respectively, *V* is the total volume of the adsorption mixture, *m* is the polymer mass, and *S* is the specific surface area of the polymer.

Binding curves were established by plotting *B* against *C* and fitting these by nonlinear regression in Origin2020Sr1 (OriginLab Corporation, USA) to a Langmuir monosite model,

$$B=\frac{B_{\max}\times K_{\mathrm{eq}}\times C}{1+K_{\mathrm{eq}}\times C}$$ 2

where *B*_max_ is the maximum amount of probe bound to each surface area unit, and *K*_eq_ is the binding constant.

Imprinting factors (IFs) were calculated by the saturated uptake ratios of the MIP and NIP, according to

$$\mathrm{IF}=\frac{B_{\max}(\mathrm{MIP})}{B_{\max}(\mathrm{NIP})}$$ 3

### Evaluation of Glyphosate Binding at Varying Salinity Levels

Materials (100 mg) were packed into the bodies of 1 mL polypropylene syringes provided with porous polyethylene retainer frits of 20 μm porosity, both from Supelco (Bellefonte, PA, US). The solid-phase extraction (SPE) cartridges thus prepared were activated by flushing with 6 mL of water at a flow rate of 2 mL/min prior to the loading step. Glyphosate (15 mL of a 20 μM solution in water) was loaded onto the packed syringes at a flow rate of 1 mL/min, followed by washing with two aliquots of 1 mL of water. The adsorbed glyphosate was eluted with three 1 mL portions of 50 mM NaCl in water. All fractions, including loading, washing, and eluting, were analyzed by ion chromatography. To evaluate the salt matrix sensitivity, the loading of glyphosate was carried out with samples of varying salinity. The 20 μM glyphosate solutions were prepared in water with sodium chloride added to practical salinity units (PSU) of 0.1, 0.2, 0.5, and 1.3, which correspond roughly to 100, 200, 500, and 1300 ppm concentration. The loading, washing, and eluting were carried out according to the above-described protocol. The recovery was calculated according to the following equation

$$\mathrm{Recovery}\;(\%)=\frac{C_{\mathrm{eluting}}\times 3}{15\times 20}$$ 4

where *C*_eluting_ is the glyphosate concentration (μM) in the three combined 1 mL eluting fractions, determined by the ion chromatography method.

### Investigating the Binding Mechanism between Glyphosate and the Imprinted Monoliths by Attenuated Total Reflection Fourier Transform Infrared (ATR-FTIR) Spectroscopy

The binding mechanisms between the imprinted monoliths and glyphosate were characterized by ATR-FTIR spectroscopy using a Vertex 70/v spectrometer from Bruker (Billerica, MA, USA) equipped with a deuterated l-alanine triglycine sulfate (DLaTGS) detector. One milliliter aliquots of 10 mM glyphosate solution were equilibrated with 5 mg of each adsorbent in 1.5 mL polypropylene microcentrifuge tubes. Water, D_2_O, and varying concentrations of NaCl (in deuterated water) to reach practical salinity units (PSU) of 0.1, 0.2, 0.5, and 1.3 were used as adsorption media. Comparison tests were conducted in the same manner using only adsorption media without added glyphosate. These tubes were then capped and shaken as above for 20 h at room temperature before centrifugation (3 min at 2800 × *g* relative centrifugal force). The supernatant was disposed of, and the adsorbent was washed with an additional 1 mL aliquot of the corresponding medium, followed by centrifugation as above. The paste-like pelleted adsorbents were transferred onto the optical window of the Golden Gate Diamond ATR accessory (Specac, Optington, UK), dried under a gentle stream of dry nitrogen gas for 1 min, and then pressed using the anvil. To minimize the effect of excess water on the quality of data, FTIR spectra were continuously collected and monitored during the evacuation until constant intensities were reached in the OH or OD stretchings (3000–3600 or 2200–2700 cm^–1^, respectively) and in the H_2_O or D_2_O bending regions (∼1650 or ∼1206 cm^–1^, respectively).^42,43^ Measurements were carried out in the 600–4000 cm^–1^ range at a resolution of 1 cm^–1^ with a 10 kHz forward/reverse scanning rate of the moving mirror. Each spectrum was obtained by coadding 100 spectra collected over a ∼356 s period. The first spectrum of the paste-like monoliths and the last spectrum of the driest achievable state of the samples on the ATR stage were used to evaluate the changes in binding mechanisms between samples. No baseline correction was applied, and all spectra were normalized to peak intensities from 0 to 1, except when a scale bar is shown in the figure. Spectra evaluation was performed with **R** version 4.1.1.^44^

### ^1^H Nuclear Magnetic Resonance (NMR) Spectroscopic Titration

A 10 mM glyphosate solution was prepared by adding 8.45 mg of glyphosate to 5.00 mL of D_2_O. In a separate vial, 25.70 mg of PMFM was added to a mixture of 5.00 mL of D_2_O and 0.30 mL of ACN-*d.* ^3^ The stock solutions of host oligomer PMFM and guest glyphosate in D_2_O were combined in NMR tubes in the following molar ratios: 0:10, 2:8, 3:7, 4:6, 5:5, 6:4, 7:3, 8:2, and 10:0. The total concentrations of the host and the guest were 2 mM, and the final volume was 0.5 mL. ^1^H NMR spectra were thereafter recorded, and the proton chemical shifts were aligned according to the chemical shift of the ACN-*d* ^3^ signal at 2.06 ppm^45^ and then used for the evaluation of the host–guest interaction.

### Material Characterizations

See the Supporting Information for description of the characterization experiments.

## Results and Discussion

### Design and Synthesis of the Molecularly Imprinted Monoliths

The chosen target glyphosate is a highly water-soluble molecule (log *K*_ow_ < −3.4), which forms ionic species of different charge with several tautomers, depending on pH.^46–49^ The melamine-based scaffold chosen for its imprinting is a cationic resin with positive charges located in the triazine ring,^50,51^ as illustrated by trimethylolated melamine, its presumed precursor (Figure S1). Our previous works^36,37^ have shown that cross-linked melamine–formaldehyde forms rebinding sites when imprinted with templates designed to represent phosphopeptides and sialylated glucans, in which phosphonate and carboxylate groups, respectively, facilitate electrostatic interactions. The melamine–formaldehyde chemistry should therefore be a good choice in an attempt to prepare monolithic MIPs targeting glyphosate.

The interactions between glyphosate and cross-linked melamine oligomers were evaluated by ^1^H NMR titration with molar ratios of glyphosate to PMFM (a commercial methylated melamine–formaldehyde oligomer) from 0:10 to 10:0 (Figure 1). The PMFM showed a signal from the −O–C**H**_**3**_ methyl protons at 3.37 ppm (Figure 1a), whereas the peaks from the −NH–C**H**_**2**_–O– and −NH–C**H**_**2**_–OH methylene protons were seen at about 4.86 and 5.13 ppm, close to a strong **H**DO peak at 4.70 ppm (Figure S2). The multipeaks at 0.92, 1.34, 1.54, and 3.62 ppm emanate from 1-butanol, the solvent of the commercial PMFM preparation used. Two signals in the ^1^H NMR spectra were assigned to glyphosate, a doublet at 3.24 and 3.27 ppm (**H2** ^2^; J_P‑‑H_ = 12.7 Hz), corresponding to the protons of the methylene group adjacent to the phosphonate group, and a singlet (**H1**) at 3.86 ppm (Figure 1a) assigned to the methylene protons next to the carboxylate group.^52,53^ A 1:1 mixture of glyphosate and PMFM in D_2_O had all corresponding peaks as components (Figure 1a).

**Figure 1:**
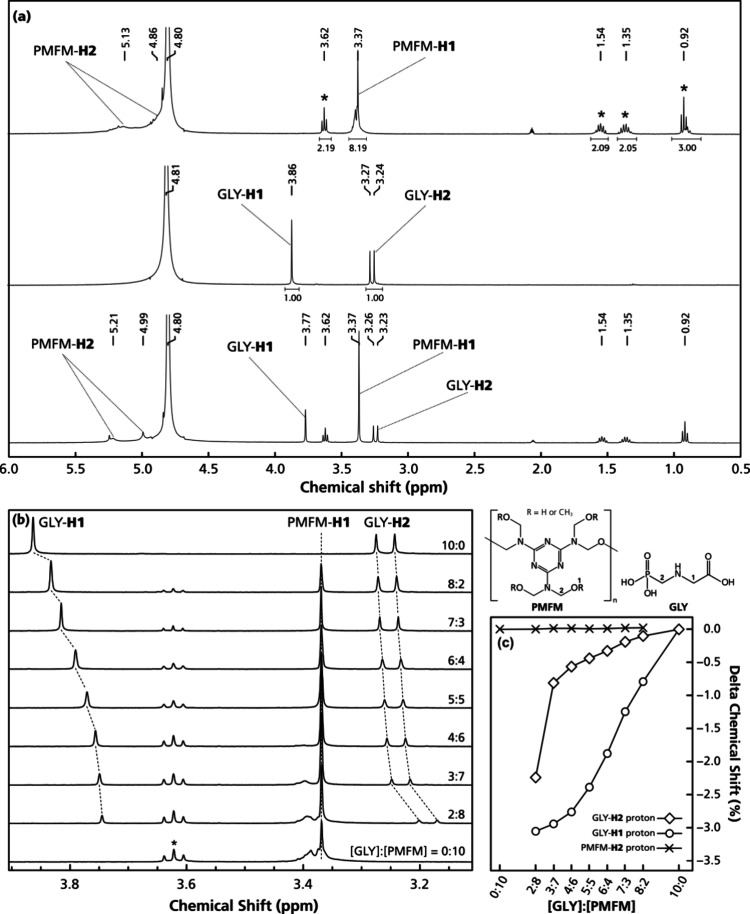
(a) ^1^H NMR spectra of (top) PMFM, (middle) glyphosate, and (bottom) their 1:1 mixture at 10 mM total concentrations in D_2_O. (b) ^1^H NMR spectra and (c) delta chemical shift of the **H1** and **H2** protons of GLY and the **H2** protons of PMFM as a function of the titration ratio between glyphosate and PMFM in D_2_O from pure glyphosate (10:0) to pure PMFM (0:10). The asterisks in panels (a) and (b) indicate a triplet signal of 1-butanol, the solvent of the commercial PMFM solution.

No shifts were seen for the PMFM-**H1** signal (Figure 1), indicating the absence of a binding site close to the −O–C**H**_3_ terminal. Nevertheless, the signals at about 4.86 and 5.13 ppm from the PMFM-**H1** protons downfield shifted by 2.8 and 2.0%, respectively (Figure S2), revealing that an association had formed, either by ion-pairing or by H/D-bonding^54–56^ of the −OH/–NH groups of PMFM toward the epitopes of glyphosate. Downfield shifting due to deshielding effects has been reported in the analysis of complex formation during imprinting prepolymerization.^24,34,57–61^

When the concentration ratio between glyphosate and PMFM was varied from 10:0 to 8:2 (Figure 1b), both of the proton NMR signals of glyphosate shifted upfield, disregarding the fast exchange between the different states on the NMR time scale, indicating an enhanced shielding effect around the methylene groups. This suggests that the glyphosate had entered into a host–guest complex with the PMFM oligomer.^62,63^ It should be noted that the titration was conducted in 100% water, an unfavorable solvent for noncovalent imprinting approaches^64–67^ due to its tendency to disrupt the hydrogen bonds in the prepolymerization complex. The use of melamine and formaldehyde, which are hydrophilic monomers with multiple polar functional groups, should be an effective solution for selective binding in protic solvents like water. Across the experimental ratio range (Figure 1c), the glyphosate protons adjacent to the carboxylate group (glyphosate-**H1**) were shifted more than those adjacent to the phosphonate group (glyphosate-**H2**), which implies a more favorable binding toward the carboxylate epitope.

The ^1^H NMR titration proved that a melamine-based polymer should be a good candidate for glyphosate imprinting, with a template to functional monomer ratio of about 1:4, in agreement with the optimum ratio of MIP synthesis.^68,69^ In our choice of template, we considered that template bleeding due to incomplete template removal from synthesized MIP^70^ is a challenge when the analyte is used as the template. We therefore used two glyphosate analogs, 4-phosphonobutanoic acid (PBA) and *N*-(phosphonomethyl)­iminodiacetic acid (PMIDA) (Figure 2), as templates to produce MIPs designated as **M-PBA1**, **M-PMIDA1**, **M-PBA0**, and **M-PMIDA0**. The last digits of the material designations, ‘1’ or ‘0’, indicate the presence or absence of formic acid (FA) as a catalyst in the monomer cocktails. Nonimprinted materials (**NIP**) were polymerized alongside the MIPs using the same monomer/porogen compositions with formic acid added but without template. The porous monolithic materials were synthesized by cross-linking a melamine–formaldehyde prepolymer by a cryopolymerization approach with a solvent/porogen mixture consisting of acetonitrile/water with mono- and triblock polyethers as structure-promoting agents, adapted from our previous works.^36,37^

**Figure 2:**
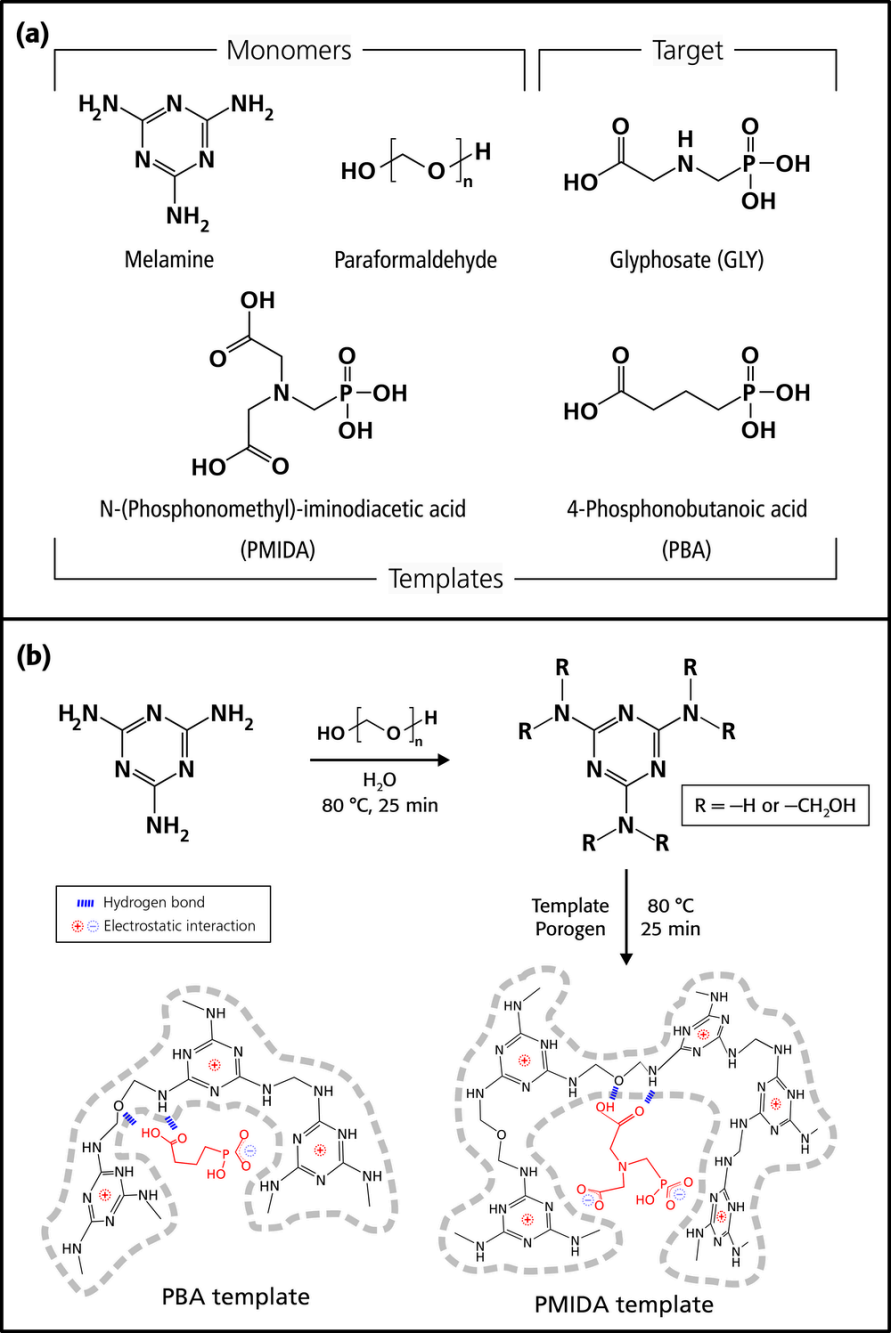
(a) Chemical structures of the target (glyphosate), the two templates (PBA, PMIDA), and the two monomers used for preparing imprinted monoliths selective toward glyphosate. (b) Synthetic route and proposed binding interaction for using PBA or PMIDA as the template.

PBA has terminal phosphonic and carboxylic acid groups spaced as in glyphosate, except that the central methylene group has been substituted for the secondary amino group in glyphosate. A geometric structure optimization was performed using the energy minimization method of Chem 3D (Revvity, Waltham, MA, USA), followed by determination of atom distances and torsion angles by Mercury (Cambridge Crystallographic Data Centre, Cambridge, UK) (Figure S3). This modeling shows that PBA has a similar distance between the carbon and phosphorus atoms of its carboxylate and phosphonate groups, in comparison with glyphosate, 5.409 and 5.107 Å, respectively. Due to the absence of the secondary amine nitrogen, PBA is straighter than glyphosate, evident from the 0.73° C1–C2–C3–P1 torsion angle. These factors combined should make PBA a suitable analogue template for monolith imprinting targeting glyphosate, with the caveat, of course, that it lacks the hydrogen-bonding abilities of the secondary amine hydrogen of glyphosate. On the other hand, PMIDA, which is a byproduct in the synthesis of glyphosate,^1^ has length and torsion angle values closer to glyphosate, 5.094 Å and 30.10°, respectively. It also provides an additional acetic acid group symmetrically bonded to the tertiary amine, which could not only promote the imprinting efficiency but also pose a steric hindrance during the imprinting.

As mentioned above, the last numbers of the MIP designations, 1 or 0, indicate the presence or absence of formic acid (FA) as a catalyst in the monomer cocktails. The polycondensation of melamine and paraformaldehyde is promoted by protons,^36,71–73^ and in this work, the smallest carboxylic acid, FA, was used in the MIPs denoted by a final ‘1’. It should be noted that both PBA and PMIDA are stronger Bro̷nsted acids than FA, with their first p*K*_a_s 3.75 for FA, 2.36 for PBA, and 0.90 for PMIDA. The polymerization reactions without FA (denoted by a final “0”) therefore served to examine the effects of having PBA and PMIDA simultaneously performing the dual roles as templates and catalysts.

FTIR spectra of dried monoliths confirm the chemical equivalency of the nonimprinted (NIP) and the four imprinted monoliths (MIPs; Figure 3). The strong and broad absorbance bands at 3440 and 3339 cm^–1^ correspond to the O–H and N–H stretching vibrations. The medium intensity peak at 2939 cm^–1^ is due to the C–H stretching vibrations, and the intense bands at 1548 and 812 cm^–1^ are from the quadrant stretching and bending of the triazine ring.^39^ A strong signal at 1481 cm^–1^ indicates the overlap of methylene C–H bending vibrations and semicircle stretching vibrations of the triazine ring.^39^ The absorbance at 1337 cm^–1^ corresponds to aromatic C–N stretching vibrations, while the 1155 cm^–1^ band arises from both aliphatic C–N and asymmetric C–O–C stretching vibrations, respectively.^39^ The peaks at 1062 and 1014 cm^–1^ are due to symmetric C–O–C and C–O stretching vibrations, respectively, while the N–H bending band at 747 cm^–1^ is assigned to the tautomeric form of the triazine ring.^39,74–77^ There were no bands in the 1640–1680 cm^–1^ region in the spectrum of the NIP, indicating the absence of −NH_2_ in the final monolith.^78^ It should furthermore be noted that no signals attributable to the glyphosate analog templates could be seen in the MIP spectra, since bands in the 1630–1740 cm^–1^ range from asymmetric stretching vibrations of the C=O motif^79–81^ present in both templates were completely absent. These results verify that the chemical compositions of the NIP and the MIPs were highly similar and prove that the templates were only involved in the imprinting process but did not take part in the polymerization.

**Figure 3:**
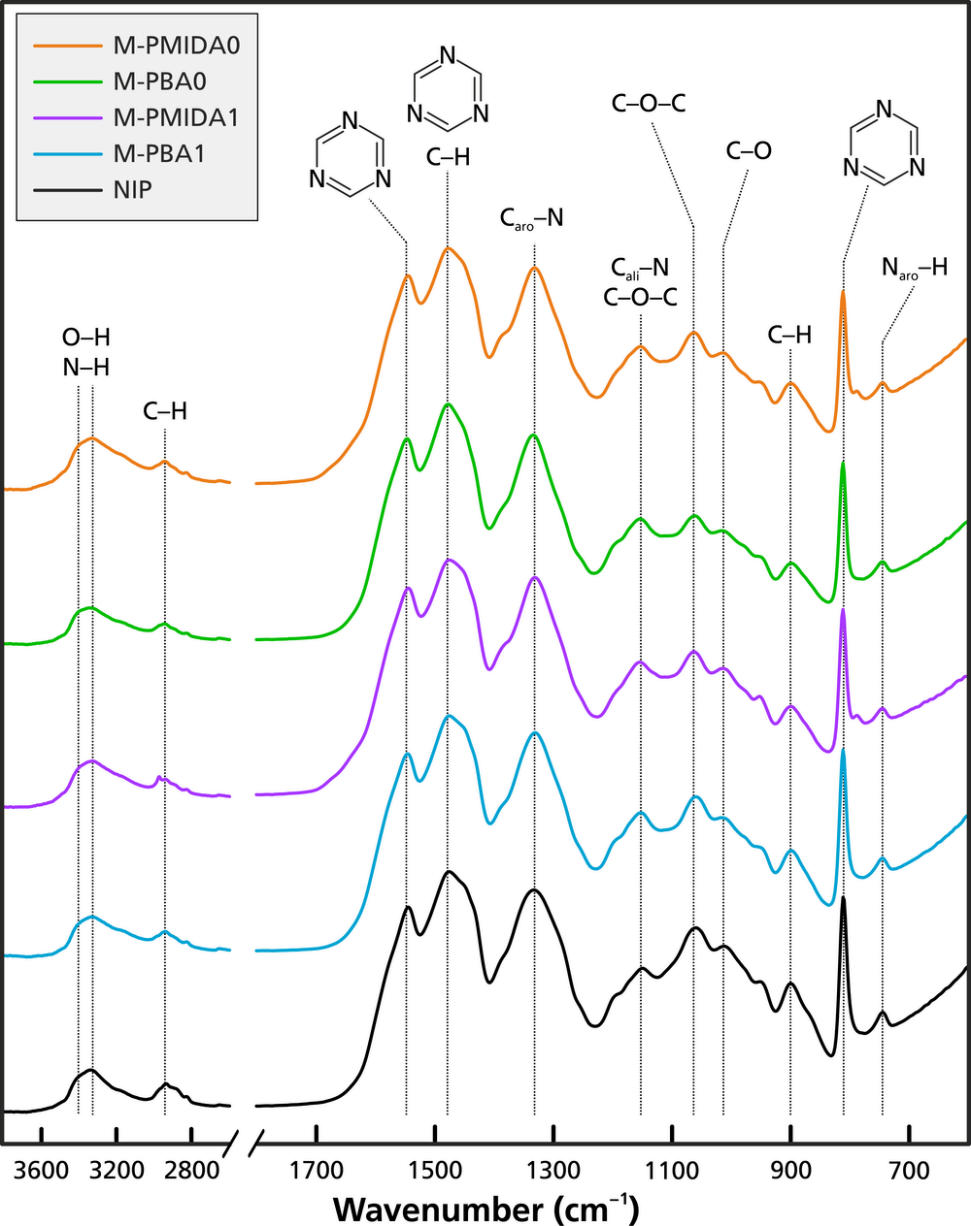
FTIR spectra of the nonimprinted (**NIP**) and the four imprinted (**M-PBA1, M-PBA0, M-PMIDA1, M-PMIDA0**) monoliths.

All monoliths had mesoporous polymer nanofiber network morphologies, as confirmed by FE-SEM (Figure S6) and nitrogen cryosorption data (Figure S7 and Table S1), with bimodal porosities consisting of 2–3 μm flow-through macropores and mesopores with median diameters in the range of 23–29 nm, as determined by the Barrett–Joyner–Halenda scheme^82^ on the desorption branch of the cryosorption data. It should be noted that the specific surface areas of these monoliths varied when different templates were used. The **NIP** and **M-PBA0** monoliths had significantly higher Brunauer–Emmett–Teller^83^ specific surface areas, with respective values of 160 and 109 m^2^/g, in comparison with 39–56 m^2^/g for other MIP monoliths.

The FE-SEM micrographs also revealed differences in the morphologies of the monoliths. All imprinted monoliths showed porous globular domains that were fused to form 3D structures, whereas a more random structure was seen in the **NIP** (Figure S6). We see two possible reasons for these differences. First, the template–monomer complex could have had an influence on the polarity of growing polymer chains, supported by the observation of the earlier onset of turbidity in the case of MIP. This is a sign of rapid formation of polymer nuclei, resulting in the creation of small, discrete globules that eventually coalesce into larger, porous domains composed of globular entities. The earlier phase separation also led to lower specific surface areas and larger pore diameters.^35,84^ Second, it cannot be ruled out entirely that the monomers or oligomers may have reacted with the templates to some extent in the polycondensation process. The latter is, however, highly unlikely due to the very low polymerization temperature (−20 °C) and the practically identical spectra without any signatures from the templates resulting from the spectral characterizations of the NIP and the MIPs (Figure 3).

### Evaluation of Glyphosate Affinity toward Imprinted Monoliths

The imprinting affinities of the MIP monoliths were assessed by the bound-free isotherm method with glyphosate as a probe in aqueous media (Figure S4 and Table 2). At first glance, the curvatures in the binding isotherms indicate specific binding sites on the imprinted monoliths, while an essentially linear increase in bound glyphosate on the nonimprinted polymer suggests that the lower density of binding sites exhibited by the NIP was nonspecific. Both the PBA and PMIDA templates resulted in imprinting of glyphosate in the hydrophilic monoliths (Table 2) as both the binding capacities and association constants of the MIPs were significantly increased compared to the NIP. With PMIDA as a template, the binding capacity for glyphosate was ≈17 μmol/m^2^ compared to ≈7.0 μmol/m^2^ for the NIP. This packing density implies that the glyphosate molecules were distributed in three dimensions on the materials. As mentioned above, PMIDA has one phosphonate and two carboxylate groups, with phosphonate to carboxylate intergroup distances and torsion angles similar to glyphosate (Figure S3). The ability of PMIDA to perform the dual roles of being both the template and catalyst was also observed for sample **M-PMIDA0**. The monolithic structure formed with this template had an anisotropic macropore system that filled the molds without giving rise to any syneresis on polymerization. In a comparison experiment, in which PMIDA was eliminated from the polymerization cocktail (a NIP without formic acid added), a monolithic structure was not formed. A small amount of solid was instead precipitated on the bottom of the reaction vial. When formic acid was used as a (co)­catalyst for **M-PMIDA1**, the specific surface area and average pore diameter of this MIP were increased but not the binding capacity.

**Table 2:** Binding Parameters from the Binding Isotherm of Glyphosate with Four Imprinted Monoliths and NIP^a^

monolith	capacity (μmol/m^2^)	association constant (M^–1^)	imprinting factor
**NIP**	7.0 ± 2.8	100 ± 54	N/A
**M-PBA1**	17.5 ± 2.6	214 ± 53	2.5
**M-PMIDA1**	17.1 ± 1.1	311 ± 39	2.4
**M-PBA0**	7.9 ± 0.93	323 ± 70	1.1
**M-PMIDA0**	17.3 ± 0.69	355 ± 28	2.5

a N/A, not applicable.

The corresponding experiments with PBA as the template showed significantly different material morphologies and binding capacities in the presence/absence of formic acid. The imprinted monolith **M-PBA0**, synthesized without formic acid catalyst, had a smaller mesopore diameter than all the other monoliths (Table S1), and a denser monolithic skeleton is also evident in the FE-SEM micrographs (Figure S6g,h). When FA was used as the catalyst (**M-PBA1**), the binding capacity was significantly increased from 7.9 to 17.5 μmol/m^2^. The synergistic effect of formic acid on the binding capacity was seen only when PBA was used as the template but not with PMIDA. The different numbers of carboxylate groups could have led to this result, as PMIDA has an extra carboxylic acid group compared to PBA, which could have acted as a catalyst for polycondensation, independent of the presence of FA.

The imprinted monolith **M-PMIDA0**, synthesized with PMIDA acting as both a template and catalyst, had the highest binding capacity and association constant (Table 2). It was therefore chosen as a model system to test the performance of the MIPs targeting glyphosate in aqueous samples of varying salinity. As described in the Materials and Methods Section, crushed monoliths were packed into 1 mL SPE syringes and activated with water, followed by loading of the test solutions containing glyphosate. Both monoliths were capable of trapping glyphosate from water (PSU = 0, Figure 4a), with high average recoveries (94–96%) after elution with 50 mM aqueous NaCl. Although the binding capacity of the imprinted monolith was significantly higher than the nonimprinted counterpart (Table 2), similar recoveries of glyphosate on **NIP** and **M-PMIDA0** were not surprising. The loading amount of glyphosate (300 ng) amounted to ≈2.5% of the capacity of the material; hence, the column would not be overloaded. The 20 μM loading solution was prepared in water so that the pH was 4.4, governed by the concentration of glyphosate and its dissociation constant. Unless the nanoporous environment of the materials considerably alters its acidity, glyphosate should, under this condition, have two negative charges on its acidic groups and a positive charge on the nitrogen atom (Figure S1). The melamine–formaldehyde polymer should, under these conditions, act as an anion exchanger,^36,85,86^ which should facilitate glyphosate adsorption onto both the imprinted sites of the **M-PMIDA0** material, as well as on the **NIP** due to nonspecific interactions.

**Figure 4:**
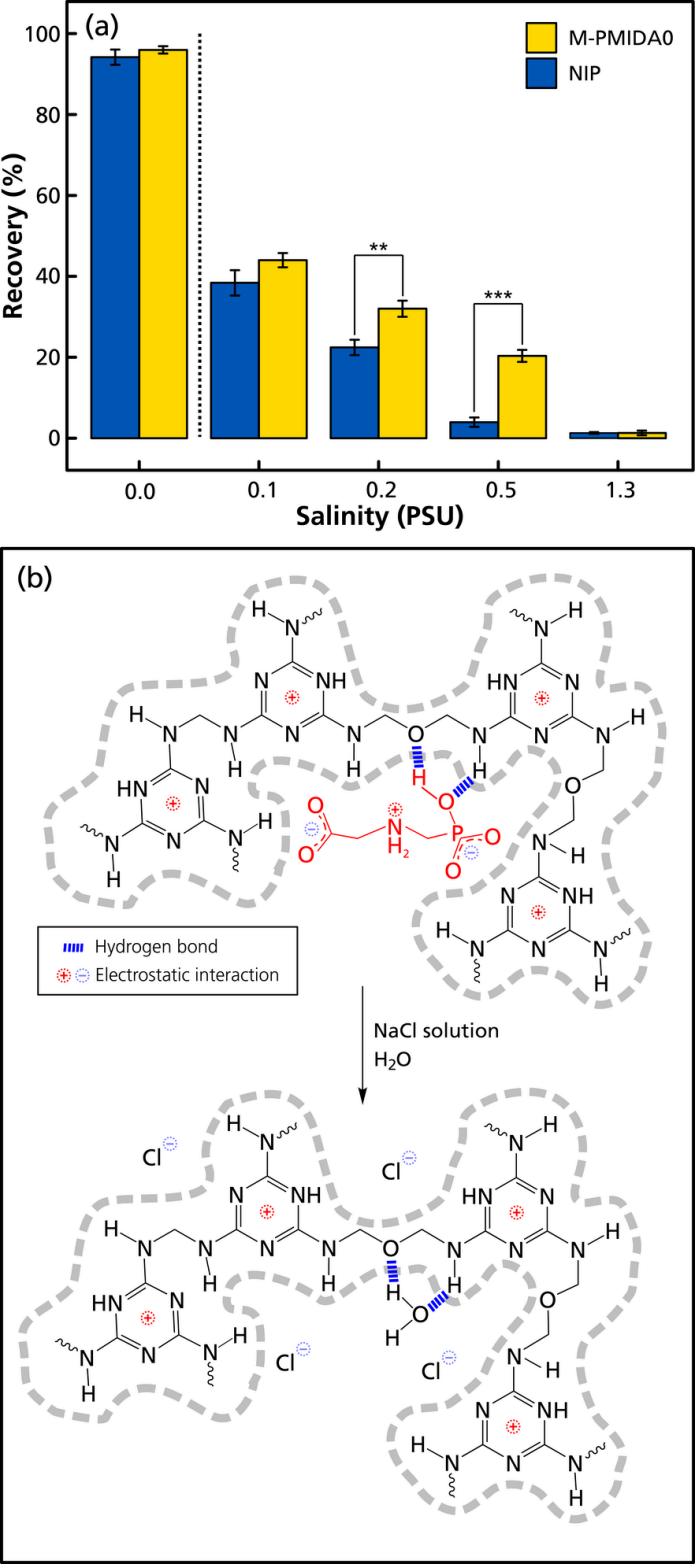
(a) Recoveries of glyphosate utilizing nonimprinted (**NIP**) and imprinted (**M-PMIDA0**) monoliths packed in SPE columns at varying salinities compared to water (*t*-test, *n* = 3: *p < 0.05, **p < 0.01 and ***p < 0.001). (b) Proposed binding with interactions between glyphosate and the imprinted monolith, and the eluting mechanism by aqueous NaCl solution.

Adsorption of glyphosate onto the imprinted monoliths was challenged by adding sodium chloride as an electrolyte to the aqueous glyphosate loading solutions by increasing ionic strength from 0.1 to 1.3 practical salinity units (PSU). The same loading, washing, and eluting procedures were applied, with the results shown in Figure 4a. The practically quantitative recovery of glyphosate from salt-free aqueous solution was severely reduced after NaCl had been added to the loading solutions from around 40% at PSU 0.1 to 1% at PSU 1.3. The presence of salt in the loading solution played the most important role in the recovery in the SPE test mode. Analyses of the loading and washing solutions, and of the eluted fractions, showed that the presence of salt had a strong negative effect on the trapping efficiency of the materials, as a substantial loss of glyphosate was seen during the loading step (Table S3). Only about 45% of the glyphosate was retained on the **NIP** in the loading step at a PSU of 0.1, gradually decreasing to 26, 7, and 1% at PSUs of 0.2, 0.5, and 1.3, respectively.

As mentioned above, the adsorption of glyphosate toward the melamine-based monoliths is based on a combination of electrostatic interactions and oriented hydrogen bonding between charged and polar groups on the glyphosate target and “pockets” with complementary functional groups imprinted on the melamine–formaldehyde monolith surfaces in the MIPs. The addition of salt triggers two competing processes simultaneously. The anionic form of glyphosate will have to compete with Cl^–^ for electrostatic binding sites on the protonated monolith surfaces by an anion exchange mechanism (Figure 4b). The second factor likely contributing to the salt-induced loss of analyte is that the added ions will shorten the Debye length by compressing the electric double layer, hence reducing the ζ-potential created by the positive charge excess on the monolith surface, which in turn lowers the potential for electrostatic interactions.^87,88^ When **M-PMIDA0** was evaluated in SPE mode, the retaining percentages at 0.2 and 0.5 PSU were 35 and 23%, respectively, which were substantially higher than for the **NIP.**

The fact that 22 mM NaCl (corresponding to a PSU of 1.3) could completely suppress the interactions between the monoliths and glyphosate shows that the main mechanism at play is electrostatic interactions, efficiently canceled by a salt screening effect.^87,88^ However, an imprinting effect was also evident as the decreases in recovery were different between the nonimprinted **NIP** and the **M-PMIDA0** imprinted materials. The recoveries of glyphosate on the imprinted monolith were 32 and 20%, which were significantly higher than those on the nonimprinted polymer (22 and 4%) under loading conditions at PSU 0.2 and 0.5, respectively (Figure 4a). This reveals that the specific binding sites toward glyphosate on the imprinted monoliths were reliant not only on nonspecific interactions but also on ligand-binding “pockets” templated to bind glyphosate, in agreement with our previous results.^89,90^

### Evaluation of Binding Interactions between Gly­phosate and Melamine-Based Monoliths by FTIR

To evaluate the binding mechanism of glyphosate on imprinted monoliths, we first compared the FTIR spectra of all monoliths before and after glyphosate adsorption. Because the IR signals of adsorbed glyphosate cannot be resolved from the dominant IR bands of monoliths, our interpretation of the binding mechanisms was based on the changes in the IR spectra of the monoliths. These changes were notably detected on the OH/NH stretching (3000–3500 cm^–1^) and the triazine- and methylene-related bands at 1481 cm^–1^ (Figure 5). Other IR bands were also evaluated; however, they remained unchanged or changed only marginally after glyphosate adsorption.

**Figure 5:**
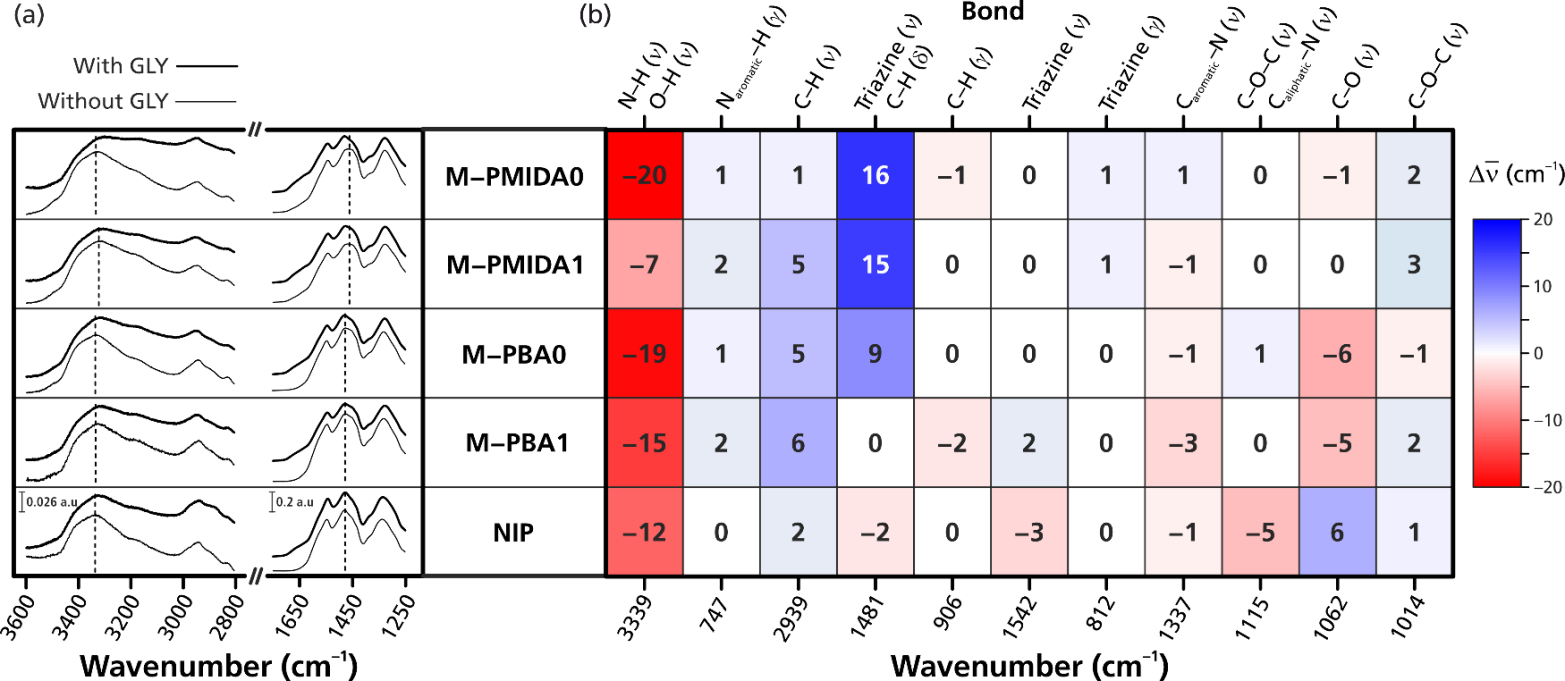
(a) Extracted FTIR spectra in the range of 3600–2800 and 1750–1250 cm^–1^. (b) Wavenumber shift heat map of the dried monoliths after 20 h immersion in an aqueous solution with and without glyphosate. Bond vibrational mode legend: ν, stretching; δ, in-plane bending; γ, out-of-plane bending.

For the first test, the adsorption of glyphosate to all five imprinted monoliths was performed in water only as the medium. The wavenumber shifts of the bands for each identifiable bond and vibration mode of the monoliths were estimated after stable signals had been reached after equilibration with and without glyphosate (Figure 5). Redshifts of up to ≈20 cm^–1^ in N–H and O–H stretching vibrations were observed for all monoliths. Additionally, the broadness of the infrared bands in the presence of glyphosate confirmed the formation of hydrogen bonds between the N–H and O–H groups with glyphosate epitopes,^78,91^ in agreement with the ^1^H NMR titration data (Figure S2). The attracting force is due to the electron affinity of X (X = N or O) in a melamine-based scaffold, which results in positively polarized H. The electron-rich O (in the carboxylate and phosphonate groups of glyphosate) is attracted by the δ^+^ hydrogen, leading to the formation of a coordinate bond. The X–H bond length is then stretched, leading to a redshift due to the decreased force constant at the atomic scale.^91^ The hydrogen bonding was present on all tested monoliths, imprinted as well as nonimprinted. This suggests the existence of induced binding sites and nonselective sites on the **NIP** surface,^92^ which, in the experiments above, showed lower affinity and capacity in comparison to the imprinted monoliths.

Significant blueshifts were noticed for methylene in-plane bending and semicircle stretching of the triazine ring, especially for MIPs using PMIDA as the template (**M-PMIDA1**, **M-PMIDA0**). In the melamine molecule, the electron cloud distribution is concentrated on the triazine ring and its nitrogen atom due to the p−π conjugation effect between the amino groups and the triazine ring. The triazine ring and its nitrogen atoms are more electronegative than the out-of-the-ring nitrogens.^93^ The inherent positive charge(s) will therefore be located in the triazine core, in agreement with the simulation (Figure S1). The bands of ν_as_(COO^–^) at 1643 cm^–1^ confirmed the negative charge state of glyphosate on both its carboxylate and phosphonate epitopes.^81^ The binding between the monolith and the anionic forms of glyphosate in an aqueous medium would hence be dominated by electrostatic interactions between the triazine core and carboxylate/phosphonate groups. The blueshift can then be attributed to the increased asymmetry of the triazine ring and its tautomeric isoform.^78,94^

Besides semicircle stretching of the triazine ring, many −CH_2_– vibrations are also likely to overlap in the fingerprint region (1410–1500 cm^–1^). In cross-linked melamine–formaldehyde materials, methylene groups act as bridges between heteroatoms in functional moieties, such as −NH–**CH**_**2**_–OH, −NH–**CH**_**2**_–O–, and −NH–**CH**_**2**_–NH–;^36,37,39,71,72,74^ hence, specific assignments are not possible. Moreover, due to obscurations among some of these methylene vibrations, small changes in peak positions cannot be assigned to moieties.^39,78^ However, the clear shifts toward higher wavenumbers for these −CH_2_– vibrations indicate changes in the chemical environment of the −CH_2_– groups upon the addition of glyphosate to the **M-PMIDA1** and **M-PIMIDA0** MIPs.^78,95^ The formation of “memory pockets” in MIPs relies on the association of the template and functional monomers in the prepolymerization reaction cocktail mediated by complementary groups in the interacting parties. Soft anions, such as the templates PBA and PMIDA, also have an affinity for moderately hydrophobic groups.^25,96^ As the MIP cocktails solidify by polymerization, selective binding sites are formed, in which the glyphosate would retain and enhance the shielding effect around its −CH_2_– groups. Another explanation for the higher wavenumbers of the methylene groups is blue-shifted bonds.^97–103^ The C–H bond was not involved in hydrogen bonding directly; however, adjacent groups, namely, −NH–, −OH, and −O–, could be HB acceptors. The strong hydrogen bonding between glyphosate and heteroatoms adjacent to the methylene bridges would trigger elongation of the C–X (X = N or O) bonds. The C–H bonds then respond to this by contraction, showing up as blueshift in vibration spectrometry.

There were also slight wavenumber shifts in the range of 1000–1150 cm^–1^ (Figure 5), which correspond to hydrogen bonding with C–O and C–O–C groups as acceptors. It should, however, be noted that these peaks are also in the range of X-sensitive modes,^78^ which makes them less useful in characterization. Moreover, these shifts vanished in the parallel tests, in which the adsorbed monoliths were paste-like (Figure S5). This could be due to disturbance of water causing a weakening of the hydrogen bonding.^64–67^ However, the shifts around 3339 and 1481 cm^–1^ both persisted, which indicates stability of the glyphosate binding toward the imprinted monoliths, even in a protic solvent like water.

Surprisingly, a blueshift was only seen for the 1481 cm^–1^ peak assigned to methylene vibrations and semicircle stretching of triazine but not at 1542 cm^–1^ for its quadrant stretching (Figure 5). Although the samples were dried by an inert gas stream, water can bind with glyphosate, a soft anion,^96^ to form a thin layer on the surface of hydrophilic oxide^104–106^ or polymer^107–109^ materials, yielding vibration signals in the 3000–3600 and 1500–1650 cm^–1^ ranges. These signals could therefore obfuscate the evaluation of important groups, such as N–H, O–H, and the triazine ring.

The glyphosate adsorption test was then performed on the highest capacity imprinted monolith, **M-PMIDA0**, using D_2_O as the solvent. The FTIR of the material was continuously recorded under a stream of dry nitrogen until the signal from O–D (2200–2700 cm^–1^) was constant (Figure 6). The persistence of the redshift of the O–H/N–H peaks (from 3440 to 3406 cm^–1^) and the blueshifts of the methylene groups, as well as the triazine ring stretching (from 1454 to 1462 cm^–1^), confirmed the association between glyphosate and the imprinted monolith. The peak assigned to the quadrant stretching of the triazine ring shifted toward a higher wavenumber in the presence of glyphosate (from 1527 to 1535 cm^–1^), which confirms the role of melamine as a useful functional monomer with positively charged functional groups for the creation of imprinting scaffolds toward glyphosate.^23^

**Figure 6:**
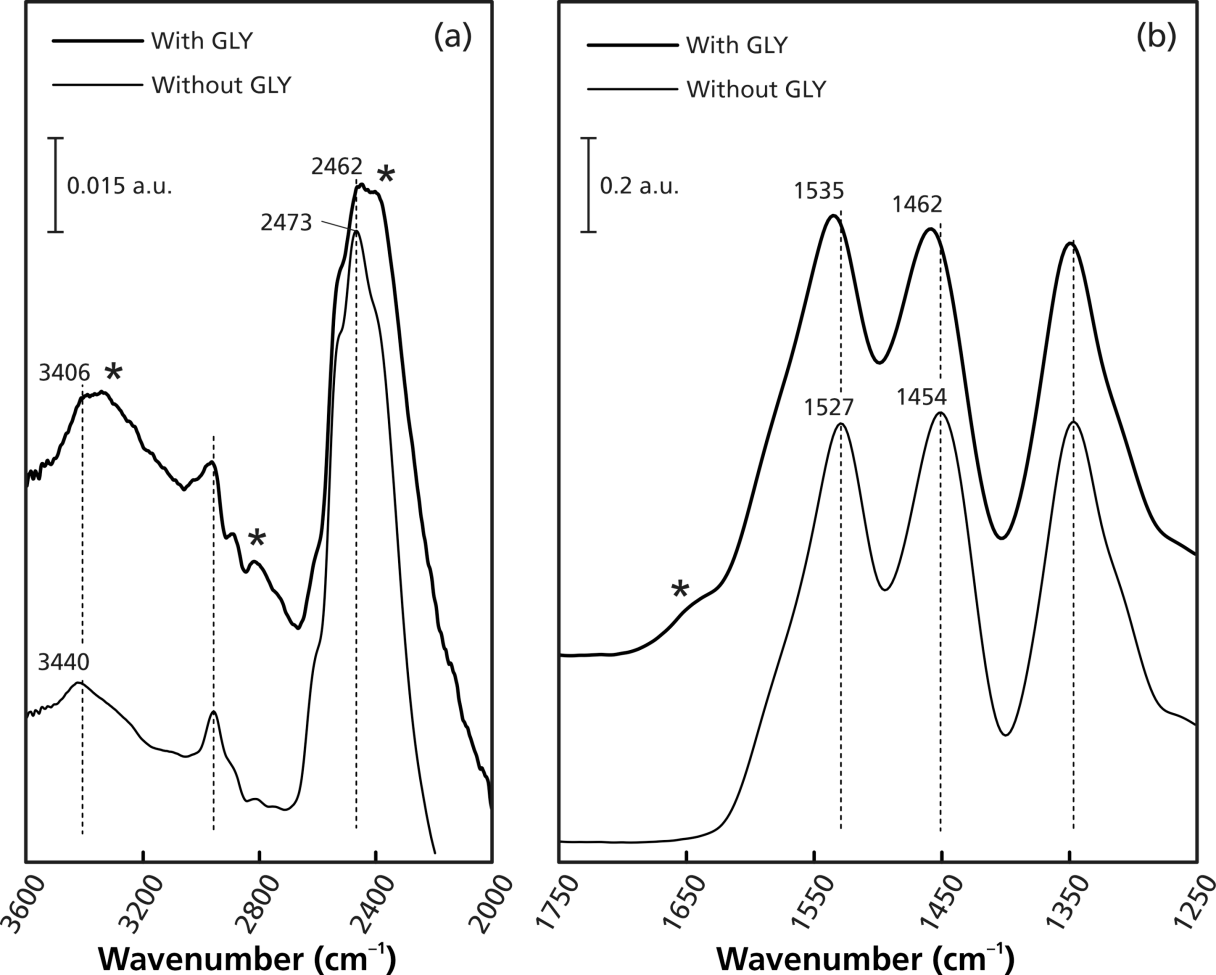
FTIR spectra (a) 2000–3600 cm^–1^ and (b) 1250–1750 cm^–1^ of the imprinted **M-PMIDA0** monolith with and without 10 mM of glyphosate in D_2_O. Asterisks indicate the signal of glyphosate.

In the section above dealing with affinity evaluation of the imprinted monoliths, the binding of glyphosate decreased when the electrolyte in the form of NaCl was added to the loading medium. In order to investigate this, from the perspective of vibration spectrometry, glyphosate solutions were prepared in D_2_O with varying NaCl contents, from PSU 0.1 to 1.3, before running the adsorption procedure with **M-PMIDA0**. The FTIR signals from the materials were monitored continuously under a stream of dry nitrogen until the signal from O–D (2200–2700 cm^–1^) was constant (Figure 7). In the presence of NaCl, the hydrogen bonding between glyphosate epitopes and the imprinted polymer was weakened, as expressed by a blueshift from 3402 to 3412 cm^–1^ of the N–H/O–H groups. The decrease in electrostatic interactions toward the triazine ring was also observed by a redshift of the two peaks at 1462 and 1535 cm^–1^. Despite most of the vibrational signals from glyphosate being too small and overlapped by substrate bands, the strong signal at 1643 cm^–1^ correlated with ν_as_(COO^–^) was noticed (Figure 7b). This peak was broad in the medium without added electrolyte but became sharper in the media with higher NaCl concentrations. It could mean that some interactions had been hindered by NaCl.^91,110,111^ These findings are fully consistent with the results from affinity evaluation experiments (Figure 4a). Because of the self-eluting effect of excess Cl^–^ toward anionic glyphosate species augmented by the salt-induced reduction of the Debye length, the reduced affinity toward glyphosate would be expected.

**Figure 7:**
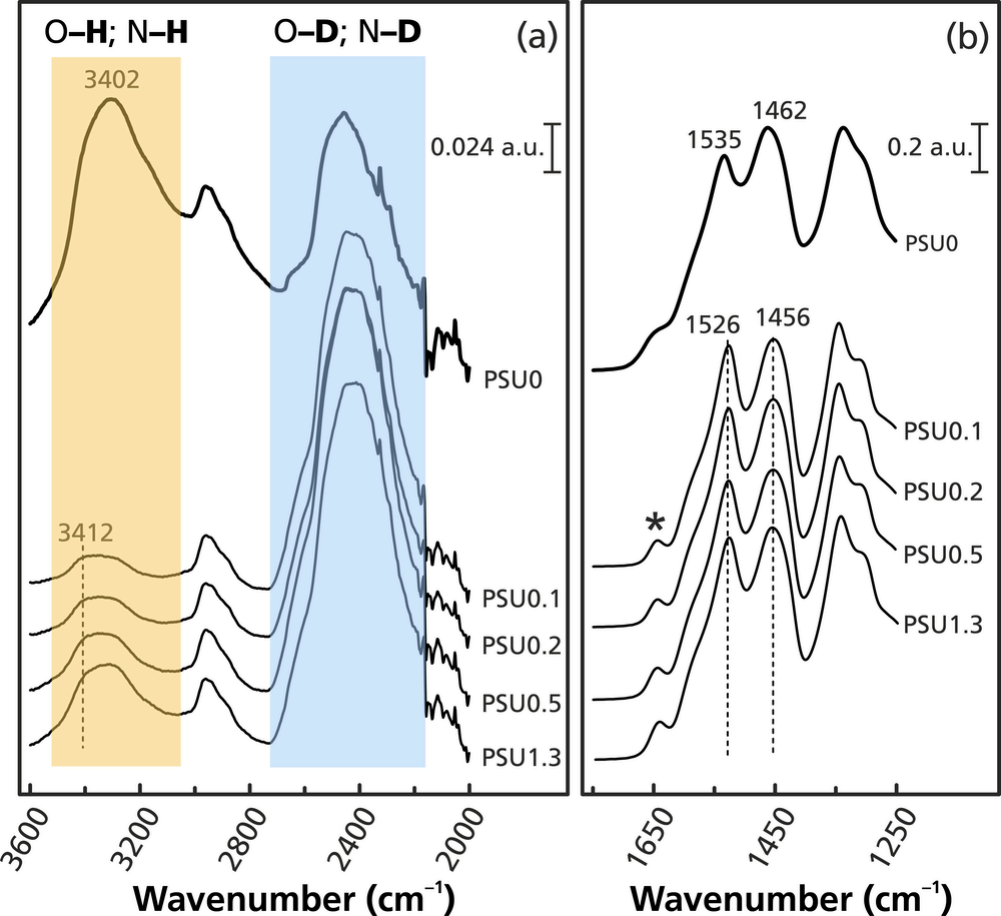
FTIR spectra: (a) 2000–3600 cm^–1^ and (b) 1250–1750 cm^–1^ of the imprinted monolith (**M-PMIDA0**) with 10 mM glyphosate in D_2_O with varying salinities. PSU: Practical Salinity Unit. The asterisks indicate a signal from glyphosate.

The band intensity ratio of X–D (X = N, O) (2200–2600 cm^–1^) over X–H (2800–3600 cm^–1^) changed radically in the presence of NaCl (Figure 7a) from 0.8 to 4.9, respectively. In other words, the hydrogen–deuterium (H–D) exchange rate on hydroxyl and amine groups of the cross-linked melamine scaffold was approximately six times faster in NaCl medium than in pure D_2_O. The relative rate of H–D exchange is correlated with the presence of hydrogen bonds,^112,113^ which confirms (i) the binding of glyphosate to functional groups in the melamine–formaldehyde monolith thanks to hydrogen bonding and electrostatic interaction and (ii) the induction of binding dissociation in the presence of electrolyte.

## Conclusions

Molecularly imprinted polymeric monoliths targeting glyphosate were prepared on melamine–formaldehyde scaffolds using 4-phosphonobutanoic acid (PBA) and *N*-(phosphonomethyl)­iminodiacetic acid (PMIDA) as templates. The assessed binding capacities and association constants verified that both PMIDA and PBA resulted in binding sites that were selective for glyphosate in aqueous medium with notable binding enhancements compared to the nonimprinted polymer, particularly in the presence of electrolytes. The mechanisms responsible for binding of glyphosate onto the imprinted monolith were unveiled by FTIR and ^1^H NMR spectroscopy, indicating the involvement of both the carboxylate and phosphonate groups of glyphosate by the simultaneous contribution of (i) electrostatic interaction toward the triazine ring and (ii) hydrogen bonding with N–H/O–H moieties, leading to the formation of selective “pockets” for glyphosate imprinted on the pore surfaces of the three-dimensional melamine–formaldehyde scaffold.

The next stage in this research could focus on optimizing the imprinting conditions, such as testing a new template, to promote surface imprinting for improved binding specificity and efficiency. Additional characterization techniques, such as solid-state NMR and cryogenic electron microscopy (Cryo-EM), could provide deeper insights into the binding mechanisms. Moreover, testing the developed materials with complex samples, such as food, soil, or wastewater, will be valuable to demonstrate the application of the product.

## Supplementary Material



## Notes

Experimental details, background information, and results for the characterization experiments (PDF)

## §

Northvolt Revolt AB, Torsgatan 122, S-931 36 Skellefteå, Sweden

## ∥

Department of Physics, Chalmers University of Technology, S-412 96 Göteborg, Sweden.

## ⊥

Klaria AB, Virdings allé 2, S-754 50 Uppsala, Sweden.
