# Supplementary material for: Melamine-Based Molecularly Imprinted Monoliths Targeting Glyphosate in Aqueous Media: Synthesis and Binding Mechanism Elucidation
Source: ACS Omega. 2025 May 23;10(22):22412–25. doi: 10.1021/acsomega.4c06690 (PMC12163837; doi:10.1021/acsomega.4c06690)
Supplement: Supplementary file 1 [file ao4c06690_si_001.pdf]

Supporting Information

Melamine-based Molecularly Imprinted Monoliths Targeting Glyphosate in Aqueous Media: Synthesis and Binding Mechanism Elucidation

Chau Minh Huynh[a], N. Tan Luong[a], Trung Nguyen[a], Ngoc Phuoc Dinh[b], Jean-François Boily[a], Knut Irgum[a*]

a) Umeå University, Department of Chemistry, S-901 87 Umeå, Sweden.

b) Diduco AB, Tvistevägen 48C, S-90736, Umeå, Sweden.

Table of Contents

Nitrogen Cryosorption ... 2
Field-Emission Scanning Electron Microscopy ... 2
Fourier-transform Infrared Spectroscopy ... 2
Ion Chromatography Method ... 2
Estimation of pK_a ... 2
Preparation of the Bjerrum Plots ... 2
Figure S1. Speciation diagrams of glyphosate and trimethylol melamine in the pH range 0 to 8 ... 2
Figure S2. ^{1}H NMR spectra from titration of glyphosate and PMFM in D_2O ... 3
Figure S3. Geometrically optimized structures of glyphosate, PBA, and PMIDA ... 3
Figure S4. Binding curves showing the amount of bound glyphosate versus free concentration ... 3
Figure S5. FTIR spectra in the 3600-2800 and 1750-1250 cm^{-1} ranges with a wavenumber shift heat map ... 4
Figure S6. Field emission scanning electron micrographs of NIP and MIP monolith cross-sections ... 5
Figure S7. Nitrogen adsorption-desorption isotherms with BJH pore size distribution inserts ... 6
Scheme S1. Ionization scheme for glyphosate with the dominant tautomers at each ionization stage ... 6
Table S1. Specific surface area, average pore, and median mesopore diameter of the monoliths ... 7
Table S2. pK_a values for formic acid, glyphosate, and the two templates PBA and PMIDA ... 7
Table S3. Glyphosate found in the loading, washing, and eluting fractions for samples of varying salinities ... 7
References ... 7
Appendix 1: Modified *speciation* and *bjerrum* functions from the *seacarb* package for R ... 8

* Corresponding author. Phone: +46 70 6444275; Email: knut.irgum@umu.se

Characterization and Evaluation Procedures

Nitrogen Cryosorption. About 100 mg of extracted and dried cubiform monolith pieces with ≈ 2 mm sides, was transferred to dry sample tubes and further dried for 2 h at 60 °C on a Micromeritics (Atlanta, GA, USA) SmartPrep degassing unit under a dry N_2 flow. The analysis was then carried out with a Micromeritics TriStar 3000 automated gas adsorption analyzer at cryoscopic temperature to obtain multipoint nitrogen adsorption-desorption data. The Brunauer-Emmett-Teller (BET) model[S1] was used to estimate the specific surface areas of the monolithic materials based on volume *vs.* pressure data from the adsorption branch in the relative pressure range from 0.18 to 0.35. Average pore diameter and median mesopore diameter were evaluated using the Barrett–Joyner–Halenda (BJH) scheme[S2] based on the desorption branches of the isotherms.

Field-Emission Scanning Electron Microscopy. Sample stubs from Ted Pella (Redding, CA, USA) were prepared with conductive tape and used to retain samples with freshly broken surfaces. A ten nanometer thick layer of Pt was thereafter coated on the sample surface using a Q150TS sputter coater from Quorum Technologies (Ringmer, UK). The coated sample was then subjected to analysis by field emission scanning electron microscopy using a Zeiss Merlin (Carl Zeiss Microscopy GmbH, Oberkochen, Germany) with an acceleration voltage of 5 kV. Images were captured from random areas at pre-determined magnifications.

Fourier-transform Infrared Spectroscopy. The NIP and MIP monoliths were characterized by ATR-FTIR spectroscopy using a Bruker Vertex 70/V spectrometer with a DLaTGS detector. Dried monoliths (≈ 10 mg) were ground manually and then pressed to the diamond of a single-bounce Attenuated Total Reflectance (ATR) accessory from Golden Gate Specac (London, UK) using the anvil provided. All measurements were carried out in the 600–4000 cm^{-1} range at a resolution of 0.5 cm^{-1}, with 10 kHz forward/reverse scanning rate of the moving mirror. Each final spectrum was obtained by co-adding 100 spectra collected over a ~ 89 s period.

Ion Chromatography. Anion chromatographic analysis was performed using an HPLC system provided with a conductivity detector from Costech (Valencia, CA, USA). Samples of 100 µL volume were separated on a 100 mm long by 4 mm i.d. Metrosep A Supp 5 column from Metrohm (Herisau, Switzerland) at 35 °C. The eluent contained 8 mM Na_2CO_3 and 2.5 mM $NaHCO_3$, delivered at a flow rate of 0.7 mL/min. The conductivity detector and set to operate at 35 °C with 2–1000 µM working range when fed the effluent from a Xenoic® XAMS chemically regenerated membrane suppressor system from Diduco (Umeå, Sweden). Chromatograms were evaluated by Clarity 2.3.0.197 from DataApex (Prague, Czech Republic).

Estimation of pK_a. The acid dissociation constants of trimethylol melamine (the MF polymer precursor), PBA, and PMIDA were estimated using the pK_a calculation package of MarvinSketch 21.13 (Chemaxon, Budapest,Hungary) with default parameters.

Preparation of the Bjerrum Plots. The Bjerrum plots in Figure S1 were prepared by extending the *bjerrum* and *speciation* functions in the *seacarb* package[S4] version 3.2.2 (run under **R** version 4.2.2[S5]) to tetravalent species. See Appendix 1 below for the modified code.

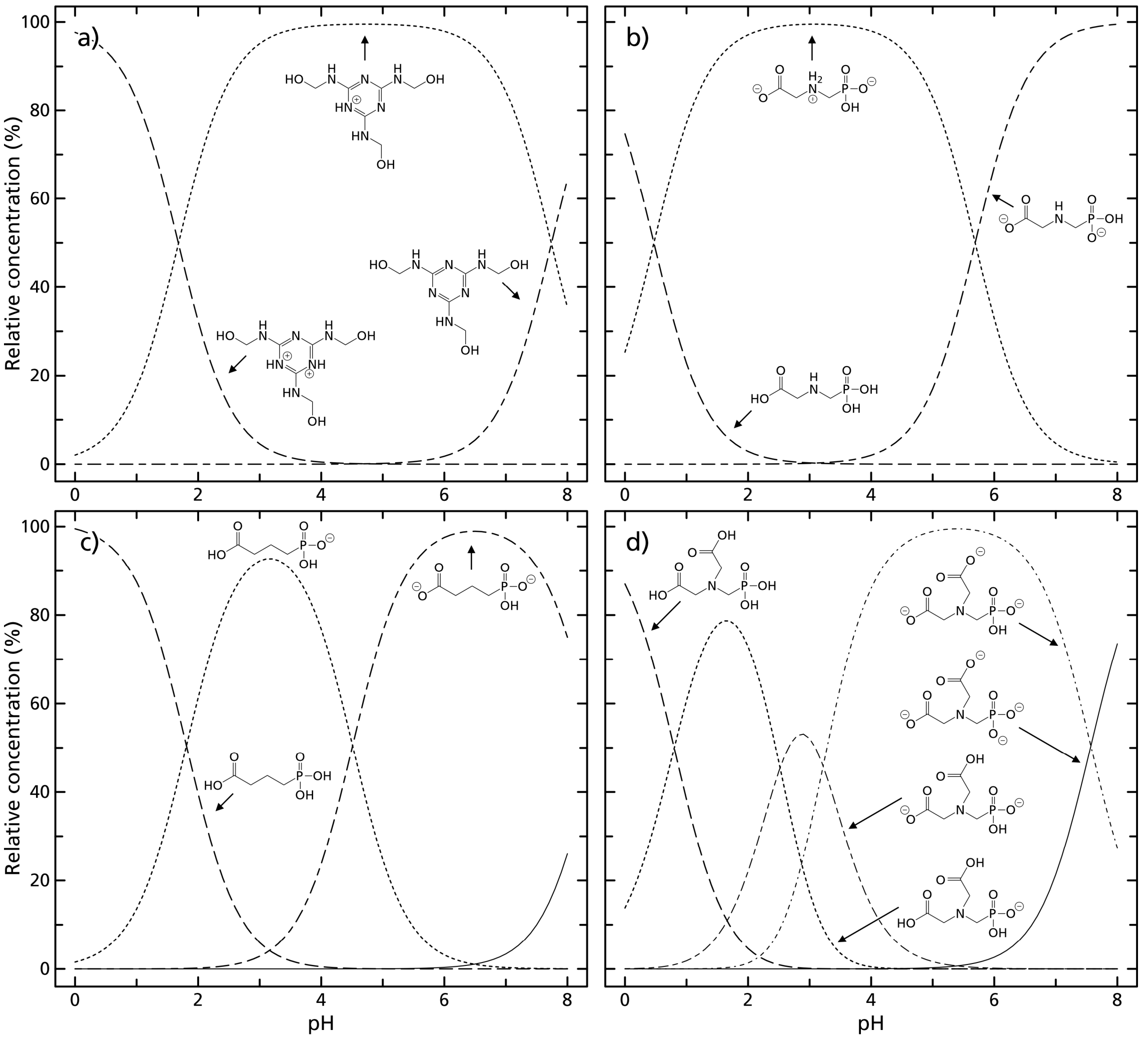

Figure S1. Speciation diagrams in water of (a) trimethylol melamine (MF prepolymer), (b) glyphosate, (c) PBA, and (d) PMIDA in the pH range 0 to 8. Glyphosate is plotted based on data from ref S3, others by data from the pK_a calculation package of MarvinSketch 21.13.

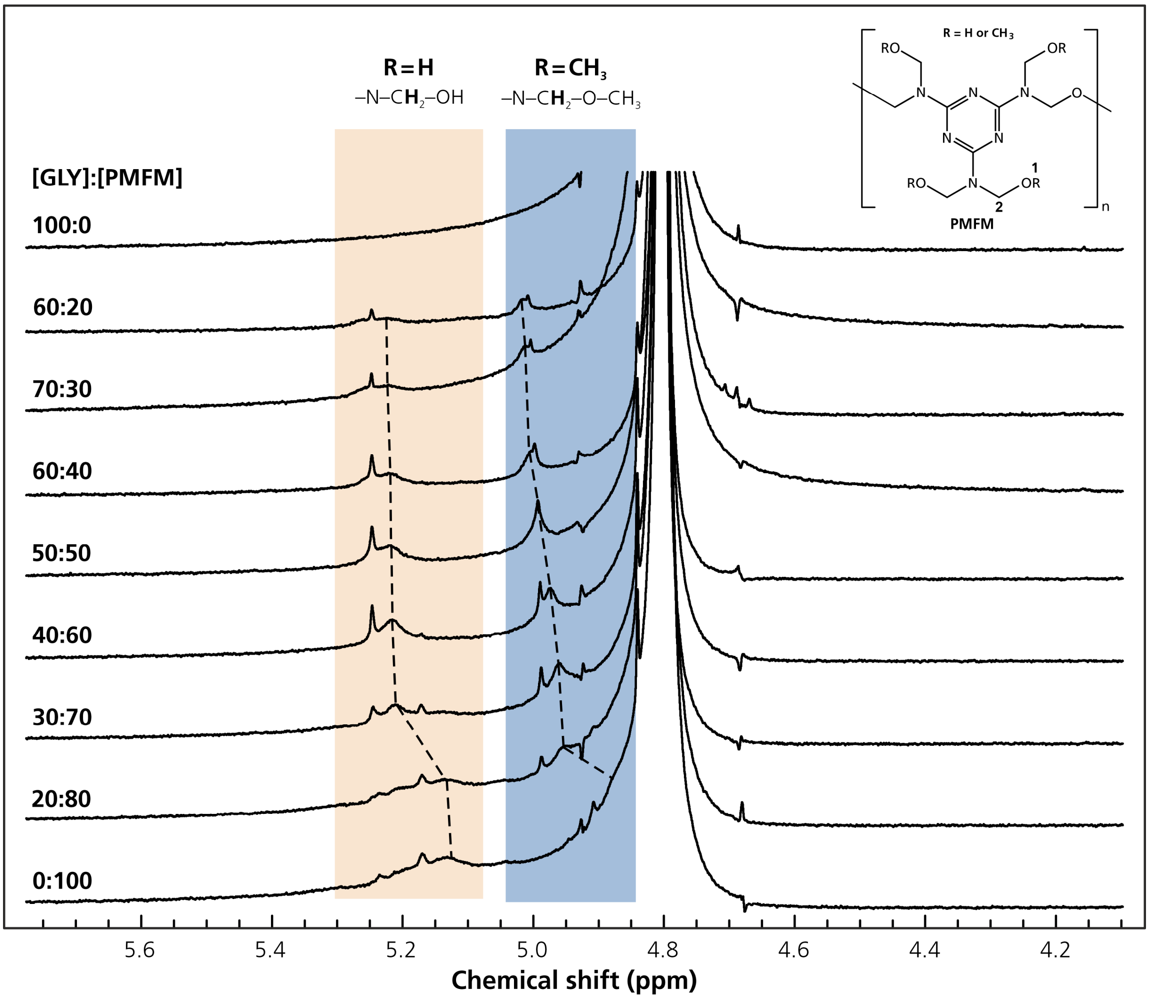

Figure S2. ^{1}H NMR spectra from titration between glyphosate and PMFM in D_2O from pure glyphosate (100:0) to pure PMFM (0:100), zoomed to the chemical shift range covering the CH_3–O–C**H**$_2$–N– and HO–C**H**$_2$–N– protons.

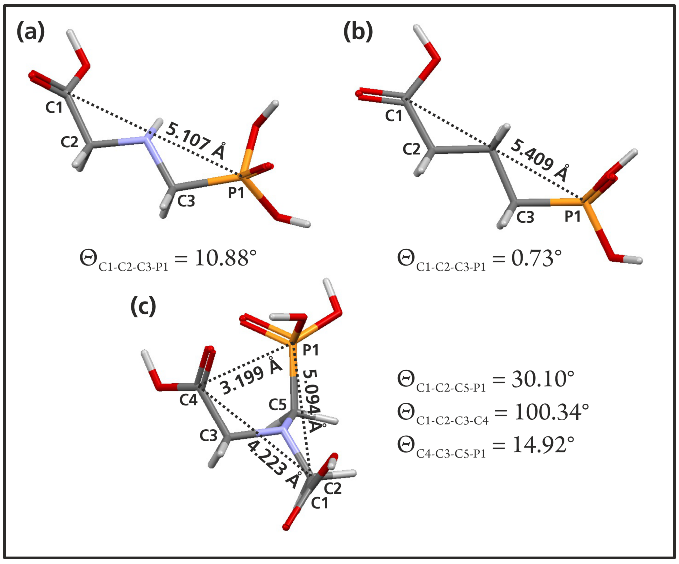

Figure S3. Geometrically optimized structures of (a) glyphosate, (b) PBA, and (c) PMIDA prepared using Chem3D with energy minimization. Atom distances and torsion angles were derived by Mercury (The Cambridge Crystallographic Data Centre).

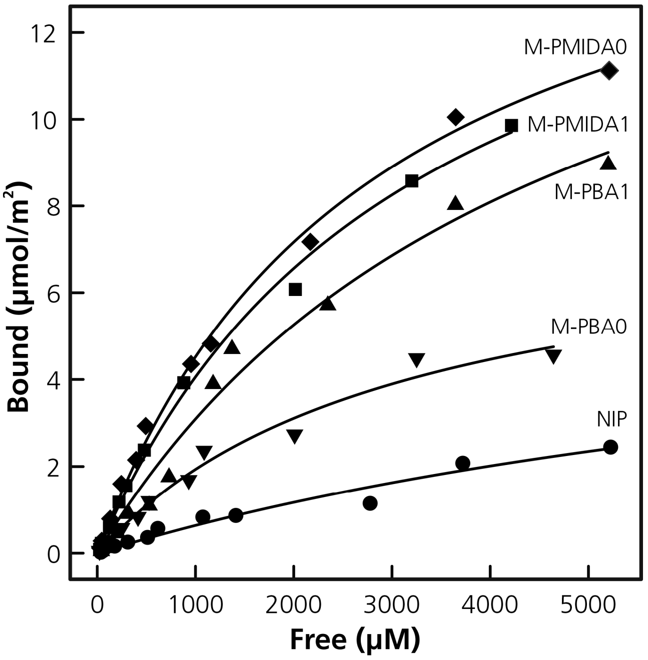

Figure S4. Binding curves showing the amount of bound glyphosate, versus free concentration after incubation with crushed non-imprinted (**NIP**) and imprinted (**M-PBA0**, **M-PBA1**, **M-PMIDA0**, and **M-PMIDA1**) monoliths. Lines in the plot are the experimental data fitted to a Langmuir mono-site model by non-linear regression.

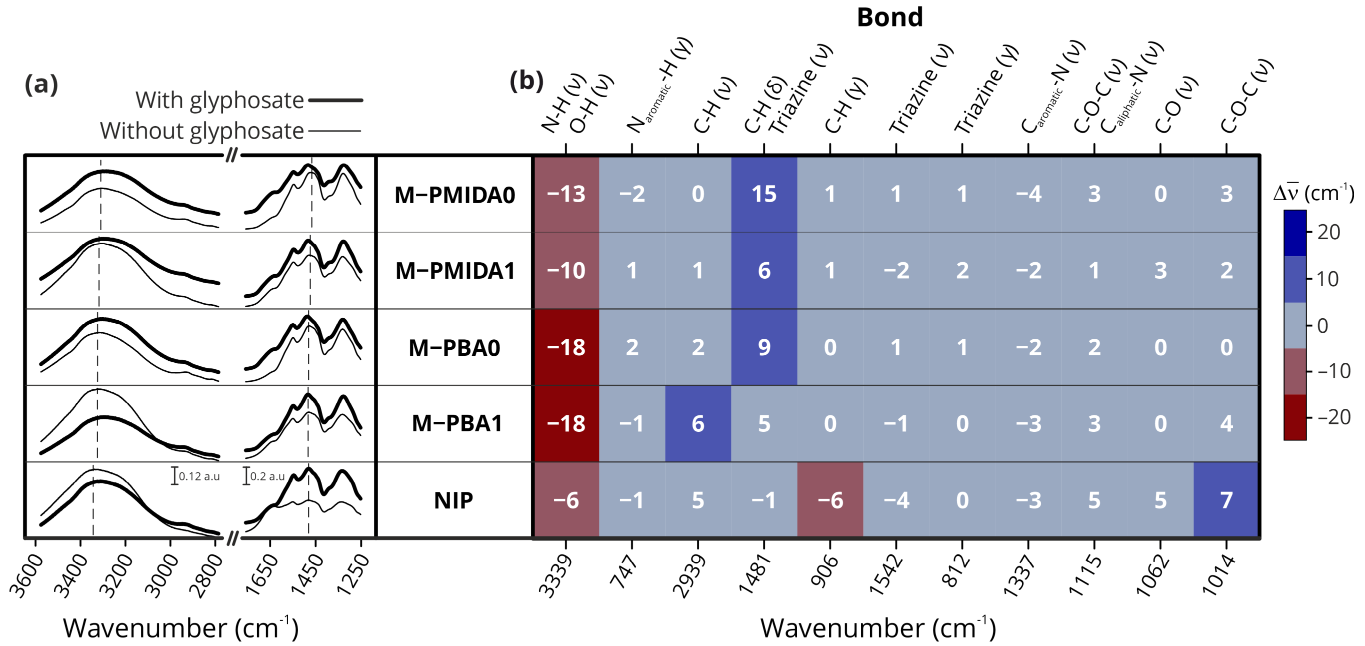

Figure S5. (a) FTIR spectra in the 3600-2800 and 1750-1250 cm^{-1} ranges; (b) wavenumber shift heat map of the paste-like monoliths after 20 h immersed in aqueous solution with and without glyphosate. ν, stretching; δ, in-plane bending; γ, out-of-plane bending.

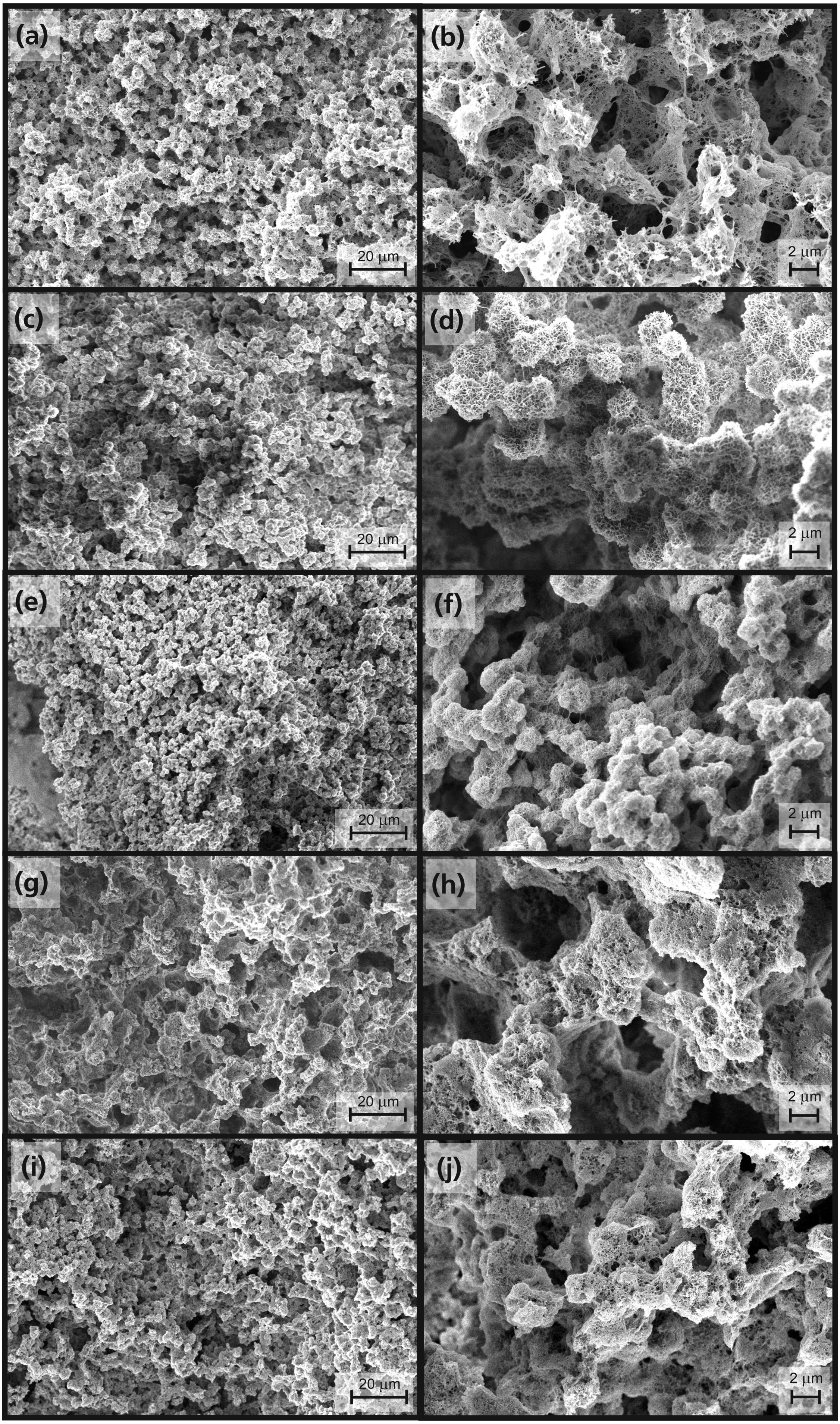

Figure S6. Field emission scanning electron micrographs (FE-SEM) of random fracture cross-section surfaces of **NIP** monolith (a, b), and MIP monoliths **M-PBA1** (c, d), **M-PMIDA1** (e, f), **M-PBA0** (g, h), and **M-PMIDA0** (i, j) at magnifications 2000 (left) and 10000 (right).

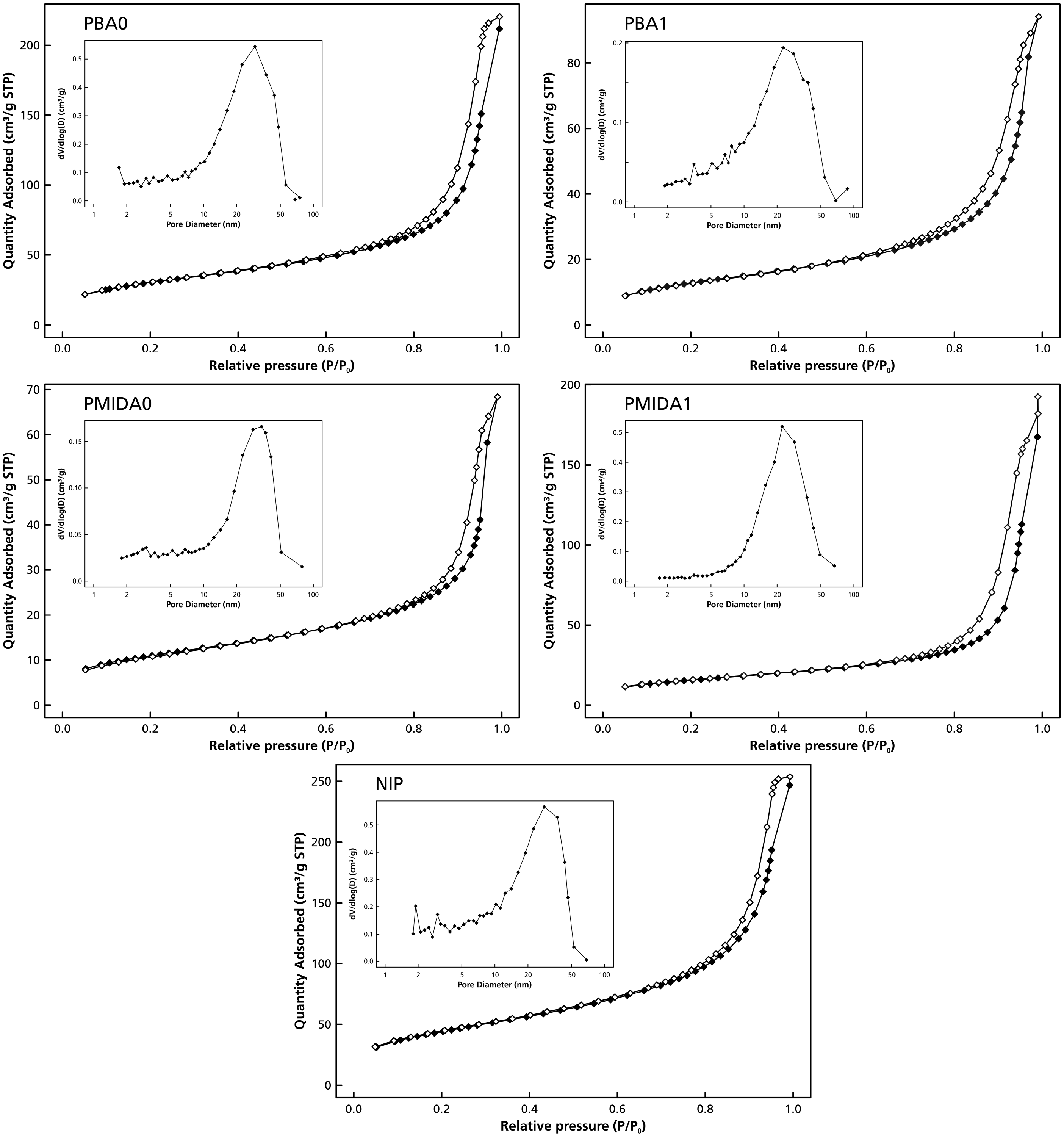

Figure S7. BET nitrogen adsorption-desorption isotherm with insert of BJH pore size distribution of (a) **NIP** monolith, and MIP monoliths (b) **M-PBA1**, (c) **M-PMIDA1**, (d) **M-PBA0**, and (e) **M-PMIDA0**.

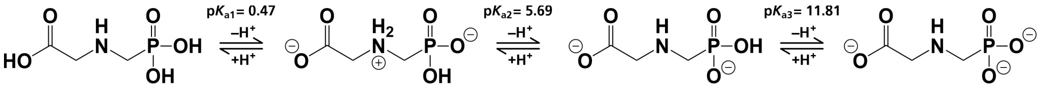

Scheme S1. Ionization scheme for glyphosate with the dominant tautomer at each ionization stage, according to ref S3.

Table S1. Specific surface area, average pore, and median mesopore diameter of the monoliths.

Monolith	Specific surface area	Average pore diameter	Median mesopore diameter
	(m^2/g)	(nm)	(nm)
**NIP**	160.2 ± 0.66	9.6	28
**M-PBA1**	46.7 ± 0.20	12	28
**M-PMIDA1**	55.8 ± 0.27	18	29
**M-PBA0**	109.1 ± 0.53	12	23
**M-PMIDA0**	39.0 ± 0.22	11	28

Table S2. Acid dissociation constants (pK_a) for formic acid, glyphosate, and the two templates PBA and PMIDA.

Compound	pK_{a1}	pK_{a2}	pK_{a3}	pK_{a4}
**FA**	3.75	—	—	—
**Glyphosate**[a)]	0.47	5.69	11.81	—
**PBA**[b)]	1.81	4.51	8.46	—
**PMIDA**[b)]	0.80	2.52	3.22	7.57

a) From ref S3. b) Predicted using MarvinSketch 21.13 (ChemAxon).

Table S3. Glyphosate found in the loading, washing, and eluting fractions of non-imprinted (NIP) and imprinted (**M-PMIDA0**) monoliths with loading solutions containing different salinities.

Salinity (PSU)	NIP			M-PMIDA0		
	Loading	Washing	Eluting	Loading	Washing	Eluting
0.0	ND	2.1 ± 0.13	94.2 ± 1.9	ND	0.97 ± 0.047	96.0 ± 0.92
0.1	55.5 ± 2.8	3.5 ± 2.00	38.4 ± 3.1	52.2 ± 1.1	3.2 ± 2.1	44.8 ± 1.8
0.2	74.4 ± 3.4	3.2 ± 0.98	22.4 ± 1.9	65.3 ± 2.1	2.5 ± 1.1	32.0 ± 2.0
0.5	92.9 ± 2.4	3.0 ± 0.82	4.0 ± 1.1	76.9 ± 1.5	3.1 ± 0.62	20.3 ± 1.5
1.3	99.1 ± 0.09	ND	1.3 ± 0.22	99.1 ± 0.09	ND	1.3 ± 0.59

Values given are in percent of the loaded amount. ND, not detected, or sample concentration is less than LOD of IC method (2.1 mM)

References

(S1) Brunauer, S.; Emmett, P. H.; Teller, E. Adsorption of Gases in Multimolecular Layers. *J. Am. Chem. Soc.* **1938**, *60*, 309–319.

(S2) Barrett, E. P.; Joyner, L. G.; Halenda, P. P. The Determination of Pore Volume and Area Distributions in Porous Substances. I. Computations from Nitrogen Isotherms. *J. Am. Chem. Soc.* **1951**, *73*, 373–380.

(S3) Peixoto, M. M.; Bauerfeldt, G. F.; Herbst, M. H.; Pereira, M. S.; da Silva, C. O. Study of the Stepwise Deprotonation Reactions of Glyphosate and the Corresponding pK_a Values in Aqueous Solution. *J. Phys. Chem. A* **2015**, *119*, 5241–5249.

(S4) https://rdrr.io/cran/seacarb; accessed 2023-11-15.

(S5) R Core Team, R: A Language and Environment for Statistical Computing. R Foundation for Statistical Computing, Vienna, Austria: **2022**. URL https://www.R-project.org

Appendix 1: Modified *speciation* and *bjerrum* functions from the *seacarb* package for R

```
############################ function Speciation: Does the actual calculations ############################
# Parameters: Dissociation constants K1...K4, the pH at which speciation is wanted, and the concentration, mol/kg
# Red colored lines below have been changed or added compared to the original code (some added comments not colored)

Speciation = function(K1 = K1(), K2 = NULL, K3 = NULL, K4 = NULL, pH = 8, conc = 1) {

  # First four helper functions are created for each of the four valence cases

  univalent = function(K1, H) {
    C1 = H/(K1+H)
    C2 = 1-C1
    return(list(C1 = C1*conc, C2 = C2*conc))
  }

  bivalent = function(K1, K2, H){
    den = H*H + H*K1 + K1*K2
    C1 = H*H/den
    C2 = H*K1/den
    C3 = 1-C1-C2
    return(list(C1 = C1*conc, C2 = C2*conc, C3 = C3*conc))
  }

  trivalent = function(K1, K2, K3, H){
    den = H*H*H + H*H*K1 + H*K1*K2 + K1*K2*K3
    C1 = H*H*H/den
    C2 = H*H*K1/den
    C3 = H*K1*K2/den
    C4 = 1-C1-C2-C3
    return(list(C1 = C1*conc, C2 = C2*conc, C3 = C3*conc, C4 = C4*conc))
  }

  tetravalent = function(K1, K2, K3, K4, H){
    den = H*H*H*H + H*H*H*K1 + H*H*K1*K2 + H*K1*K2*K3 + K1*K2*K3*K4
    C1 = H*H*H*H/den
    C2 = H*H*H*K1/den
    C3 = H*H*K1*K2/den
    C4 = H*K1*K2*K3/den
    C5 = 1-C1-C2-C3-C4
    return(list(C1 = C1*conc, C2 = C2*conc, C3 = C3*conc, C4 = C4*conc, C5 = C5*conc))
  }

  # Estimate the speciation of the various ionic forms as a function of pH using the appropriate function above

   if (is.null(K1)) return()                        # Just to make sure (checked already in the Bjerrum function)
   H = 10^(-pH)
   if (is.null(K2))      res = univalent  (as.double(K1), H)
   else if (is.null(K3)) res = bivalent   (as.double(K1), as.double(K2), H)
   else if (is.null(K4)) res = trivalent  (as.double(K1), as.double(K2), as.double(K3), H)
   else                  res = tetravalent(as.double(K1), as.double(K2), as.double(K3), as.double(K4), H)
   return(res)
}

############################ function Bjerrum: Create a Bjerrum plot ############################

# Parameters: The dissociation constants K1...K4, the pH range and increment, the concentration,
# mol/kg, overruled default plotting options, "add" (false: start new, true: add to current),
# and "..." (plotting options passed to matplot)

Bjerrum = function(K1 = K1(), K2 = NULL, K3 = NULL, K4 = NULL, phmin = 2, phmax = 12, by = 0.1, conc = 1,
  main = NULL, type = "l", col = "black", ylab = "Relative concentration (%)", add = FALSE, ...) {

  # Create a Bjerrum plot
   if (is.null(K1)) return()                        # Not much to do here…

   pH = seq (phmin, phmax, by = 0.01)               # Prepare the pH gradient
   res = Speciation(K1, K2, K3, K4, pH, conc)       # Calculate the entire speciation matrix using Speciation()

   if (!add) matplot(pH, as.data.frame(res), ylab = ylab, type = type, main = main)
   else matlines(pH, as.data.frame(res))
}

windows(record = TRUE)                              # Prepare to overlay plots for scrolling (see below)
pHmin = 0; pHmax = 8                                # Set min and max pH for the plots

Bjerrum(K1 = 10^-1.68, K2 = 10^-7.74, phmin = 0, phmax = pHmax, main = "Trimethylolated melamine", lty = 1)
Bjerrum(K1 = 10^-0.47, K2 = 10^-5.69, K3 = 10^-11.81, phmin = pHmin, phmax = pHmax, main = "Glyphosate", lty = 1)
Bjerrum(K1 = 10^-1.81, K2 = 10^-4.51, K3 = 10^-8.46, phmin = pHmin, phmax = pHmax, main = "PBA", lty = 1)
Bjerrum(K1 = 10^-0.80, K2 = 10^-2.52, K3 = 10^-3.22, K4 = 10^-7.57, phmin = pHmin, phmax = pHmax, main = "PMIDA", lty = 1)

# Click on the graphics pane and use PgUp and PgDn to scroll between the four result plots
```
